# Baseline and longitudinal grey matter changes in newly diagnosed Parkinson’s disease: ICICLE-PD study

**DOI:** 10.1093/brain/awv211

**Published:** 2015-07-14

**Authors:** Elijah Mak, Li Su, Guy B. Williams, Michael J. Firbank, Rachael A. Lawson, Alison J. Yarnall, Gordon W. Duncan, Adrian M. Owen, Tien K. Khoo, David J. Brooks, James B. Rowe, Roger A. Barker, David J. Burn, John T. O’Brien

**Affiliations:** 1 1 Department of Psychiatry, University of Cambridge, UK; 2 2 Wolfson Brain Imaging Centre, University of Cambridge, UK; 3 3 Institute of Neuroscience, Newcastle University, Newcastle, UK; 4 4 Medicine of the Elderly, Western General Hospital, Edinburgh, UK; 5 5 Brain and Mind Institute, University of Western Ontario, London, Canada; 6 6 Department of Psychology, University of Western Ontario, London, Canada; 7 7 Griffith Health Institute and School of Medicine, Griffith University, Gold Coast, Australia; 8 8 Division of Brain Sciences, Imperial College London, London, UK; 9 9 Department of Clinical Medicine, Positron Emission Tomography Centre, Aarhus University, Denmark; 10 10 Department of Clinical Neurosciences, University of Cambridge, Cambridge, UK; 11 11 Medical Research Council, Cognition and Brain Sciences Unit, Cambridge, UK; 12 12 Behavioural and Clinical Neuroscience Institute, University of Cambridge, Cambridge, UK; 13 13 John van Geest Centre for Brain Repair, University of Cambridge, Cambridge, UK

**Keywords:** Parkinson’s disease, neurodegeneration, neuroimaging, dementia

## Abstract

Mild cognitive impairment in Parkinson’s disease is associated with progression to dementia (Parkinson’s disease dementia) in a majority of patients. Determining structural imaging biomarkers associated with prodromal Parkinson’s disease dementia may allow for the earlier identification of those at risk, and allow for targeted disease modifying therapies. One hundred and five non-demented subjects with newly diagnosed idiopathic Parkinson’s disease and 37 healthy matched controls had serial 3 T structural magnetic resonance imaging scans with clinical and neuropsychological assessments at baseline, which were repeated after 18 months. The Movement Disorder Society Task Force criteria were used to classify the Parkinson’s disease subjects into Parkinson’s disease with mild cognitive impairment (*n = *39) and Parkinson’s disease with no cognitive impairment (*n = *66). Freesurfer image processing software was used to measure cortical thickness and subcortical volumes at baseline and follow-up. We compared regional percentage change of cortical thinning and subcortical atrophy over 18 months. At baseline, cases with Parkinson’s disease with mild cognitive impairment demonstrated widespread cortical thinning relative to controls and atrophy of the nucleus accumbens compared to both controls and subjects with Parkinson’s disease with no cognitive impairment. Regional cortical thickness at baseline was correlated with global cognition in the combined Parkinson’s disease cohort. Over 18 months, patients with Parkinson’s disease with mild cognitive impairment demonstrated more severe cortical thinning in frontal and temporo-parietal cortices, including hippocampal atrophy, relative to those with Parkinson’s disease and no cognitive impairment and healthy controls, whereas subjects with Parkinson’s disease and no cognitive impairment showed more severe frontal cortical thinning compared to healthy controls. At baseline, Parkinson’s disease with no cognitive impairment converters showed bilateral temporal cortex thinning relative to the Parkinson’s disease with no cognitive impairment stable subjects. Although loss of both cortical and subcortical volume occurs in non-demented Parkinson’s disease, our longitudinal analyses revealed that Parkinson’s disease with mild cognitive impairment shows more extensive atrophy and greater percentage of cortical thinning compared to Parkinson’s disease with no cognitive impairment. In particular, an extension of cortical thinning in the temporo-parietal regions in addition to frontal atrophy could be a biomarker in therapeutic studies of mild cognitive impairment in Parkinson’s disease for progression towards dementia.

## Abstract (precis)

Mild cognitive impairment in Parkinson’s disease (PDMCI) is associated with progression to dementia in a majority of patients. Mak *et al.* reveal accelerated cortical thinning in patients with PDMCI compared to non-cognitively impaired patients and healthy controls. Patterns of cortical thinning may constitute biomarkers for increased dementia risk.

## Graphical abstract

**Figure:**
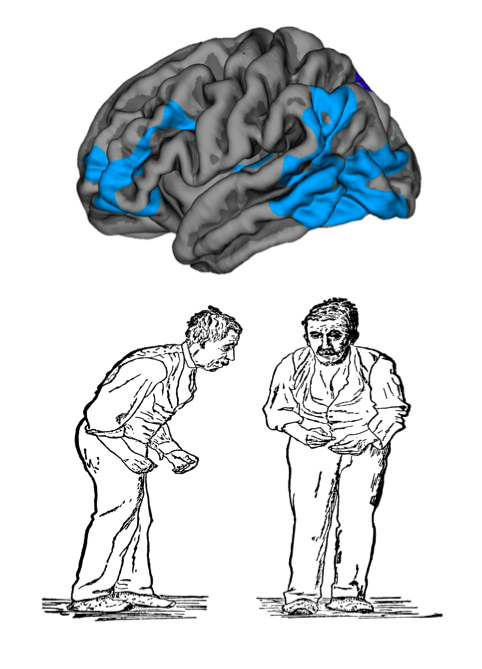
Mild cognitive impairment in Parkinson’s disease (PDMCI) is associated with progression to dementia in a majority of patients. Mak *et al.* reveal accelerated cortical thinning in patients with PDMCI compared to non-cognitively impaired patients and healthy controls. Patterns of cortical thinning may constitute biomarkers for increased dementia risk.

## Introduction

Parkinson’s disease is a progressive neurodegenerative disorder affecting over 4 million people worldwide above the age of 50, with a prevalence that is expected to double to 9.3 million by 2030 (Dorsey *et al.*, 2007). There has been a gradual shift in the definition of Parkinson’s disease, from a classical akinetic-rigid movement disorder to a complex multi-system neurodegenerative disease affecting multiple cognitive domains (Yarnall *et al.*, 2014) with debilitating consequences for patients (Lawson *et al.*, 2014) and caregivers (Aarsland *et al.*, 1999). Cognitive deficits are frequent comorbidities in Parkinson’s disease, with up to 80% of patients with Parkinson’s disease eventually developing mild cognitive impairment (PD-MCI) and dementia (Parkinson’s disease dementia) (Aarsland *et al.*, 2003).

The neural substrates of cognitive impairment in Parkinson’s disease remain only partially understood and underscore the need to establish imaging biomarkers that are capable of aiding the identification of ‘at risk’ patients for dementia and by so doing help in their early diagnosis and treatment. Previous voxel-based morphometric (VBM) studies have found detected atrophy in the temporal, parietal and frontal cortices in PD-MCI compared to Parkinson’s disease with no cognitive impairment (PD-NC) (Song *et al.*, 2011; Melzer *et al.*, 2012). These anatomical changes were mirrored by neurocognitive functional abnormalities (Nombela *et al.*, 2014). At a subcortical level, reduced volumes of the hippocampus, thalamus and the nucleus accumbens have also been reported in PD-MCI (Weintraub *et al.*, 2011; Mak *et al.*, 2014). However, a consensus concerning the presence of grey matter atrophy in PD-MCI remains to be established (Apostolova *et al.*, 2010; Dalaker *et al.*, 2010; Hattori *et al.*, 2012). A previous VBM study with a large sample of 148 subjects with Parkinson’s disease did not reveal any significant grey matter atrophy in PD-MCI compared with healthy controls (Pereira *et al.*, 2012). It is possible that these negative findings may reflect inadequate sensitivity of VBM for detecting subtle cortical atrophy in the early stages of Parkinson’s disease (Jubault *et al.*, 2011). Alternatively it could be that the different types of PD-MCI have differing neurobiological substrates (Williams-Gray *et al.*, 2009). Finally, surface-based analyses of cortical thickness may prove to be more sensitive than VBM (Pereira *et al.*, 2012), as there is evidence from corticometry studies showing cortical thinning in temporal and parietal regions in PD-MCI relative to PD-NC (Pagonabarraga *et al.*, 2013; Pereira *et al.*, 2014; Segura *et al.*, 2014).

The search for potential imaging biomarkers of cognitive impairment in Parkinson’s disease has also been hindered by a scarcity of longitudinal evidence. Beyond inherent challenges in interpretations of causality, cross-sectional measurements may be less sensitive as subtle changes tend to be masked by large interindividual variability in brain size and structure, which could in turn account for the inconsistent findings. With serial imaging, the subject serves as his or her own reference point, allowing the investigation of the spatio-temporal progression of cortical thinning to be quantified at the individual level, and in turn, provide insights into the vulnerability of certain brain regions as well as their trajectory of atrophy in the disease course of Parkinson’s disease.

In this study, we have investigated regional cortical thickness and subcortical volumes in a large and well-characterized cohort of non-demented subject with Parkinson’s disease. Subjects were classified into PD-MCI and PD-NC using the Movement Disorders Society (MDS) diagnostic criteria (Litvan *et al.*, 2012). Importantly, we evaluated the progression of cortical thinning and subcortical atrophy using serial MRI over 18 months. We hypothesized that PD-MCI would be characterized by significantly reduced cortical thickness at baseline and develop a more severe pattern of cortical thinning compared to PD-NC and healthy controls over time.

## Materials and methods

### Participants

Between June 2009 and December 2011, patients with newly diagnosed Parkinson’s disease from community and outpatient clinics in the North East of England were recruited (*n = *128) and followed up for 18 months. Over the course of the study, 18 subjects with Parkinson’s disease did not return for follow-up (15 withdrawn and three deceased) and two subjects were excluded due to a change in their diagnosis, who did not differ significantly from included Parkinson’s disease subjects in terms of age and Unified Parkinson’s Disease Rating Scale (UPDRS). Only subjects (*n = *108) with baseline and follow-up assessments were included in this study. Written requests for notification of patient details were sought from all general practitioners, neurologists, geriatricians and Parkinson’s disease specialist nurses. Parkinson’s disease was diagnosed by a movement disorders specialist according to the UK Brain Bank criteria (Hughes, 2002). Full inclusion and exclusion criteria have been previously described (Khoo *et al.*, 2013; Nombela *et al.*, 2014; Yarnall *et al.*, 2014). Specifically, subjects with dementia at presentation (DSM IV criteria for dementia or MDS criteria for dementia) were excluded. Additionally, subjective cognitive decline and functional independence of participants were determined through semi-structured interviews with participants and/or their carers for the classification of dementia as well as PD-MCI. Unrelated healthy controls (*n = *50) of similar age and sex to patients were recruited from community sources to control for normal ageing. Twelve healthy controls did not return for follow-up (11 withdrawn and one deceased) who were not significantly different in age. This resulted in a final sample size of 38 healthy controls with both baseline and follow-up scans.

### Standard protocol approvals, registrations and patient consents

The study was approved by the Newcastle and North Tyneside Research Ethics Committee. All subjects provided written informed consent.

### Clinical assessment

Clinical and demographic data were collected, including disease duration, level of education, medication and family history. Clinical assessments were performed by trained examiners, and included a standardized neurological examination, the Geriatric Depression Scale (Yesavage *et al.*, 1982), the revised Unified Parkinson’s Disease Rating Scale (UPDRS III) (Goetz *et al.*, 2008), and Hoehn and Yahr staging (Hoehn and Yahr, 2001).

### Neuropsychological assessment

In line with MDS Task Force recommendations (Litvan *et al.*, 2012), five cognitive domains were assessed. Attention was measured using the Cognitive Drug Research computerized battery (Wesnes *et al.*, 2002). Mean response times of simple reaction time, choice reaction time, and digit vigilance were summed to produce a Power of Attention score. Digit vigilance accuracy was also evaluated as part of this domain. Memory was assessed with Pattern Recognition Memory (PRM), Spatial Recognition Memory (SRM), and Paired Associates Learning (PAL) from the computerized Cambridge Neuropsychological Test Automated Battery (CANTAB) (Fray and Robbins, 1996). Executive function was determined using the modified ‘One Touch Stockings’ (OTS) version of the Tower of London task from the CANTAB battery, phonemic fluency (words beginning with ‘F’ in 1 min) and semantic fluency (animals in 90 s). The pentagon copying item of the Mini-Mental State Examination (MMSE) was graded using a modified 0 to 2 rating scale as a measure of visuospatial function. Language domain was assessed using the naming (0–3) and sentence (0–2) subsets of the Montreal Cognitive Assessment (MoCA) test.

All participants were assessed ‘ON’ their usual dopaminergic medication at baseline and 18 months. Levodopa equivalent daily dose value was calculated using the Tomlinson *et al.* (2010) formula. Global cognitive function was assessed using the MMSE (Folstein *et al.*, 1975) and the MoCA (Dalrymple-Alford *et al.*, 2010). Performance on the individual tasks was transformed into z-scores. Subsequently, a composite summary index for each cognitive domain was derived from the corresponding averages of the respective neuropsychological tests. A subject was diagnosed as PD-MCI if they were impaired (1.5 SDs) below normative means on two tests in one cognitive domain or on one test in two different domains (i.e. impairment on any two tests). Consistent with previous studies (Pereira *et al.*, 2014; Yarnall *et al.*, 2014), modified level 2 criteria were used as our neuropsychological battery predated the publication of the PD-MCI criteria, in that only one test was specific to the visuospatial domain. Within the Parkinson’s disease group, 40 subjects were classified as PD-MCI while the remaining Parkinson’s disease subjects were classified as PD-NC (*n = *68).

### MRI acquisition

Subjects underwent both baseline and repeat MRI with an 18-month interval. Both MRI acquisitions were done on the same 3 T MRI system (Intera Achieva scanner, Philips Medical Systems). The structural scans were acquired using a standard T_1_-weighted volumetric sequence covering the whole brain: 3D magnetization-prepared rapid gradient echo sequence (MPRAGE), sagittal acquisition, echo time = 4.6 ms, repetition time = 9.6 ms, inversion time 1250 ms, flip angle = 8°, SENSE factor = 2, in-plane field of view 240 × 240 mm yielding a voxel size of 1.15 × 1.15 mm with slice thickness of 1.2 mm.

### Preprocessing of baseline and longitudinal imaging data

Cortical reconstruction and volumetric segmentation of MRI data was performed using the Freesurfer 5.3 image analysis suite (http://surfer.nmr.mgh.harvard.edu/) using standard methods (Fischl *et al.*, 1999; Fischl and Dale, 2000). The initial processing of T_1_ MRI images, for each subject and each time point (baseline and follow-up), includes the following steps: removal of non-brain tissue, automated Talairach transformation, segmentation of the subcortical white matter and deep grey matter volumetric structures, intensity normalization, tessellation of the grey matter/white matter boundary, automated topology correction and surface deformation to optimally place the grey matter/white matter and grey matter/CSF boundaries. The cortical thickness was calculated as the closest distance from the grey/white matter boundary to the grey/CSF boundary at each vertex.

For the longitudinal processing, an unbiased within-subject template (Reuter and Fischl, 2011) was created using robust, inverse consistent registration between the two time points (Reuter *et al.*, 2010). Several processing steps, such as skull stripping, Talairach transformations, atlas registration, as well as spherical surface maps and parcellations were initialized with common information from the within-subject template, significantly increasing reliability and statistical power (Reuter *et al.*, 2012). To facilitate the comparison of our findings, the cortical thickness maps were smoothed using a 15 mm full-width half-maximum Gaussian that is also consistent with the majority of recent cortical thickness studies in Parkinson’s disease (Ibarretxe-Bilbao *et al.*, 2012; Pereira *et al.*, 2012, 2014; Garcia-Diaz *et al.*, 2014; Segura *et al.*, 2014). All surface models in our study were visually inspected for accuracy and manual corrections were performed in the event of tissue misclassification/white matter errors while blinded to diagnostic group information. Subjects who had excessive pial/white matter surface segmentation errors after the manual correction were excluded from the subsequent baseline (one healthy control, two PD-NC, one PD-MCI) and follow-up (three PD-NC) statistical analyses.

### Baseline and longitudinal comparisons of cortical thickness

Baseline comparisons between groups were assessed using a vertex-wise general linear model (GLM). The model included cortical thickness as a dependent factor and diagnostic group (healthy controls, PD-NC, and PD-MCI) as an independent factor. Analyses were performed to determine the association between regional cortical thickness and cognitive function. For the longitudinal analyses of cortical thinning, vertex-wise comparisons of per cent change of cortical thickness among the diagnostic groups were analysed using the longitudinal two-stage GLM in Freesurfer (Reuter *et al.*, 2012). In the longitudinal analysis, the per cent change of cortical thickness was the dependent factor and the diagnostic group was the independent factor. We further investigated the association between regional cortical thickness and longitudinal changes in global cognition and the composite scores for specific cognitive domains. In all GLM analyses, age, gender and education were included as nuisance covariates while levodopa equivalent daily dose was an additional covariate in the comparisons between PD-NC and PD-MCI groups. Consistent with previous methodologies (Ibarretxe-Bilbao *et al.*, 2012; Garcia-Diaz *et al.*, 2014; Segura *et al.*, 2014), family wise error (FWE) cluster-based correction using Monte Carlo simulations with 10 000 iterations was applied to cortical thickness maps to correct for multiple-comparisons and results were thresholded at a corrected *P*-value of 0.05 (Hagler *et al.*, 2006).

### Volumetric analysis of subcortical structures

In addition, the following subcortical structures at both time-points were automatically segmented from each hemisphere using Freesurfer: thalamus, caudate, putamen, pallidum, hippocampus, amygdala, and the nucleus accumbens. Consistent with previous methodology (Mak *et al.*, 2015), we first calculated the absolute difference in volumes between both times [volume_follow-up_ − volume_baseline_] for each subject before dividing by the volume at baseline [volume_follow-up_ − volume_baseline_] / volume_baseline_ to normalize the amount of atrophy with respect to baseline. This was then multiplied by 100 to derive a percentage change score: [volume_follow-up_ − volume_baseline_] / volume_baseline_ × 100%. Subsequently, group differences in percentage change of subcortical volumes were interrogated with analysis of covariance (ANCOVA) while controlling for age, gender, education and the average of total intracranial volumes at both time points. *Post hoc* Tukey-Kramer pairwise comparisons were subsequently performed between each group. Associations of subcortical volumes with cognitive measures were also assessed with correlational tests at baseline and at follow-up.

### Statistical analyses

Statistical analyses were performed with the STATA13 (http://www.stata.com/) software. The distribution of continuous variables was tested for normality using the Skewness-Kurtosis test and visual inspection of histograms. Parametric data were assessed using either *t*-tests or analysis of variance (ANOVA) for continuous variables. For non-parametric data, Kruskal-Wallis was used. *χ*^2^ tests were used to examine differences between categorical measures. For each test statistic, a two-tailed probability value of <0.05 was regarded as significant.

## Results

### Demographics and clinical variables

The demographic and clinical information for Parkinson’s disease and control subjects are summarized in Table 1. PD-MCI subjects were significantly older than PD-NC subjects (*P = *0.001), although there were no significant differences in age between PD-MCI and healthy controls (*P = *0.170) or between PD-NC and healthy controls (*P = *0.308). Groups were well-matched in terms of gender (*P = *0.251). Years of education were significantly lower in PD-MCI compared to healthy controls (*P = *0.002) and PD-NC (*P < *0.001). Compared to PD-NC, levodopa equivalent daily dose intake was significantly higher in PD-MCI (*P < *0.001) at baseline but there were no significant differences in levodopa equivalent daily dose intake at follow-up and changes of dosage over 18 months. In addition, there were no significant differences in disease duration, Hoehn and Yahr staging or UPDRS III scores at baseline as well as change scores over 18 months (Table 1). There was a main effect of group on depression scores (Geriatric Depression Scale) [*F*(2 139) = 8.78, *P < *0.001]. *Post hoc* Tukey pair-wise tests showed that both PD-NC (*P = *0.002) and PD-MCI (*P < *0.001) groups had significantly higher Geriatric Depression Scale scores than healthy controls although there was no significant difference in mean Geriatric Depression Scale scores between PD-NC and PD-MCI. As would be expected, there was a main effect of group on MMSE [*F*(2 139) = 17.79, *P < *0.009] and MoCA scores [*F*(2 127) = 29.67, *P < *0.001] at baseline. *Post hoc* Tukey pair-wise tests revealed that PD-MCI scored significantly poorer on both MMSE and MoCA compared to PD-NC (*P < *0.001) and healthy controls (*P < *0.001). There was no difference in MMSE and MoCA between PD-NC and healthy controls. There was a main effect of group on MMSE change scores [*F*(2 135) = 4.94, *P = *0.001] over 18 months. The decline in MMSE scores was significantly greater in PD-MCI compared to PD-NC (*P = *0.038) and healthy controls (*P = *0.010). PD-NC did not significantly differ from healthy controls in terms of MMSE decline (*P = *0.677). There were no significant differences in change in the MoCA among the groups [*F*(2 124) = 1.54, *P = *0.218].

**Table 1:** Baseline and longitudinal demographics and clinical characteristics

	Healthy controls	PD-NC	PD-MCI	*P*-value
*n*	37	66	39	
Age (years)	65.7 ± 7.2	62.9 ± 9.9	69.4 ± 8.8	0.002^a^
Age range	49.7–85.4	41.8–87.3	48.1–85.5	
Gender (male, %)	21 (56.7)	41 (62.1)	29 (74.4)	0.3 ^h^
Education (years)	13.9 ± 3.9	13.8 ± 3.5	11.6 ± 3.5	0.001^e^
Disease duration (months)		24.2 ± 4.6	24.9 ± 5.1	0.5^f^
Levodopa equivalent daily dose				
Baseline		143.1 ± 110.1	248.7 ± 152.6	<0.001^f^
Follow-up		391.1 ± 202.1	470.2 ± 209.9	0.059^g^
Change		237.8 ± 213.9	221.5 ± 216.0	0.5^f^
Hoehn and Yahr				
Baseline		1.9 ± 0.7	2.1 ± 0.6	0.1^g^
Follow-up		2.1 ± 0.6	2.2 ± 0.4	0.2^f^
Change		0.2 ± 0.6	0.1 ± 0.6	0.2^f^
UPDRS III				
Baseline		25.3 ± 10.9	29 ± 10.9	0.1^g^
Follow-up		31.1 ± 12.7	37.9 ± 9.4	0.001^f^
Change		6.6 ± 10.7	8.9 ± 10.1	0.3^g^
MMSE				
Baseline	29.4 ± 1.0	29.1 ± 0.8	28.1 ± 1.4	<0.001^a^^,b,c^, 0.4^d^
Follow-up	29.6 ± 1.0	29.1 ± 1.0	27.4 ± 2.0	<0.001^a^^,b,c^, 0.1^d^
Change	0.2 ± 0.7	0.0 ± 1.1	−0.6 ± 1.9	0.009^a^, 0.010^b^, 0.038^c^, 0.7^d^
MoCA				
Baseline	27.6 ± 2.2	26.9 ± 2.4	23.1 ± 3.6	<0.001^a^^,b,c^, 0.4^d^
Follow-up	27.9 ± 3.0	27.8 ± 2.0	24.1 ± 3.5	<0.001^a^^,b,c^, 0.9^d^
Change	0.3 ± 2.7	1.1 ± 1.9	1.2 ± 3.1	0.2^a^
Geriatric Depression Scale				
Baseline	1.0 ± 1.5	2.5 ± 2.5	2.9 ± 2.1	<0.001^a^^,b^, 0.6^c^, 0.002^d^
Follow-up	1.2 ± 2.0	2.6 ± 2.9	3.2 ± 2.6	<0.001^a^, 0.004^b^, 0.5^c^, 0.042^d^
Change	0.2 ± 1.8	0.0 ± 2.7	0.2 ± 1.9	0.8^a^
Scan interval (years)	1.7 ± 0.1	1.5 ± 0.1	1.5 ± 0.0	<0.001^a^^,b,d^, 0.6^c^

Values expressed as mean ± 1SD.

^a^ANOVA = healthy controls, PD-NC, PD-MCI.

*Post hoc* Tukey pairwse tests: ^b^PD-MCI versus healthy controls; ^c^PD-MCI versus PD-NC; ^d^ PD-NC versus healthy controls.

^e^Kruskal-Wallis test.

^f^Wilcoxon rank-sum test = PD-NC and PD-MCI.

^g^Student’s *t*-test – PD-NC and PD-MCI.

^h^χ^2^ = PD-NC, PD-MCI, Controls.

### Clinical and imaging comparisons between stable Parkinson’s disease subjects and converters

Of the 66 PD-NC subjects, there were 17 converters classified as PD-MCI at follow-up. We compared demographics, clinical characteristics, and cortical thickness measures between those who remained cognitively stable (PD-stable) and those who converted to PD-MCI (PD-converters). The PD-NC subgroups did not significantly differ in age (*P = *0.129), gender (*P = *0.157), UPDRS III (*P = *0.480), and levodopa equivalent daily dose intake (*P = *0.292) at baseline. However, PD-converters performed significantly less well on the MoCA (*P = *0.039) and the MMSE at trend level (*P = *0.063) compared to the PD-stable group. At baseline, we observed a bilateral pattern of cortical thinning affecting the temporal regions in the PD-converters relative to the PD-stable subjects. However, these significant findings did not survive after correcting for age, gender, education, and levodopa equivalent daily dose intake because of low power.

### Baseline and longitudinal cortical thinning

#### PD-MCI subjects versus healthy controls

**Figure 1:**
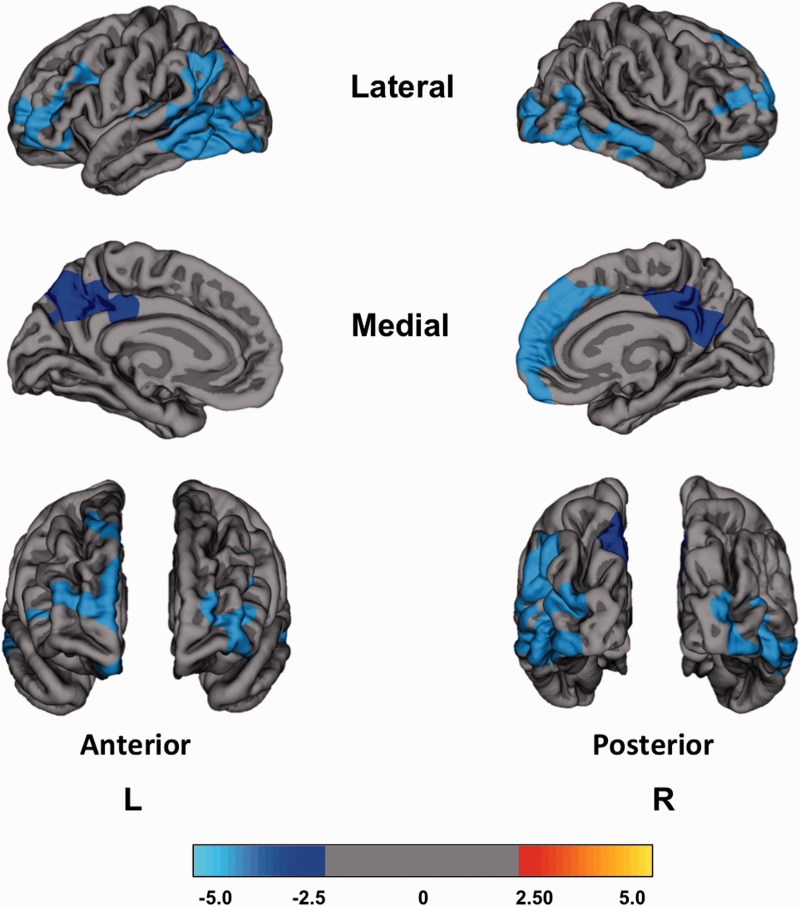
**Reduced regional cortical thickness in PD-MCI compared to healthy controls at baseline.** No significant differences in baseline cortical thickness were found between PD-MCI and PD-NC, and between PD-NC and healthy controls. The colour bar shows the logarithmic scale of *P*-values (−log_10_).

**Figure 2:**
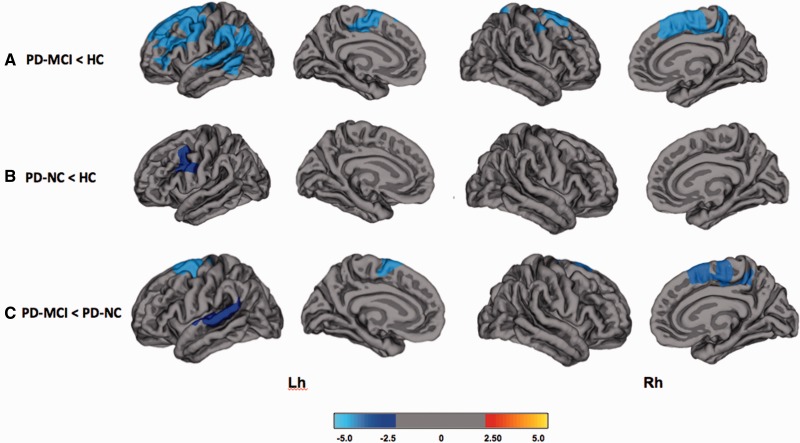
**Vertex-wise comparisons of percentage change in cortical thinning over 18 months.** (**A**) PD-MCI < HC; (**B**) PD-NC < HC; (**C**) PD-MCI < PD-NC. The colour bar shows the logarithmic scale of *P*-values (−log_10_). Lh = left hemisphere; Rh = right hemisphere.

At baseline, subjects with PD-MCI showed significantly reduced cortical thickness in the frontal, parietal and occipital cortices: left supramarginal cortex, bilateral rostral middle frontal cortex, left isthmus cingulate and right posterior cingulate cortices, and the right lateral occipital cortex (Fig.1 and Supplementary Table 1; *P < *0.05; FWE Monte Carlo cluster-wise corrected). Over 18 months, subjects with PD-MCI had significantly increased percentage of cortical thinning predominantly in the frontal and parietal cortices: left superior frontal cortex, left supramarginal cortex, and right precuneus (Fig. 2 and Supplementary Table 2; *P < *0.05; FWE Monte Carlo cluster-wise corrected).

#### PD-NC subjects versus healthy controls

At baseline, there were no significant differences in regional cortical thickness between PD-NC and healthy controls. Over 18 months, however, PD-NC subjects developed a significantly greater percentage of cortical thinning in the left caudal middle frontal cortex (Fig. 2 and Supplementary Table 2; *P < *0.05; FWE Monte Carlo cluster-wise corrected).

#### PD-MCI versus PD-NC subjects

At baseline, there were no significant differences in regional cortical thickness between PD-MCI and PD-NC. Over 18 months, compared to the PD-NC group, the PD-MCI subjects showed a significantly greater percentage of cortical thinning in the frontal and temporal cortices including the left caudal middle frontal, right superior frontal cortex and left superior temporal cortex. Compared to PD-MCI, no increased percentage changes of cortical thinning were found in the PD-NC group (Fig. 2 and Supplementary Table 2; *P < *0.05; FWE Monte Carlo cluster-wise corrected).

#### Clinical and cognitive associations of cortical thinning

**Figure 3:**
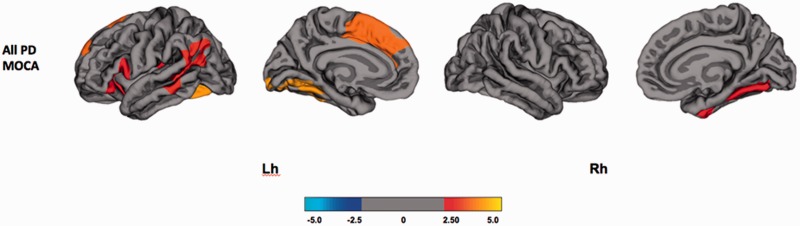
**Association between cognitive measures and regional cortical thickness at baseline.** The colour bar shows the logarithmic scale of *P*-values (−log_10_**)**. PD = Parkinson’s disease; Lh = left hemisphere; Rh = right hemisphere.

Within the combined group of non-demented Parkinson’s disease (PD-NC and PD-MCI), analyses with the MoCA revealed a significant positive association with baseline cortical thickness, indicating that increased cortical thickness was correlated with higher MoCA scores, in frontal and temporo-parietal cortices: left fusiform gyrus, left superior frontal cortex, left inferior parietal cortex, left orbitofrontal cortex and right parahippocampal gyrus (Fig. 3 and Supplementary Table 4; *P < *0.05; FWE Monte Carlo cluster-wise corrected).

#### Baseline and longitudinal comparisons of subcortical atrophy in Parkinson’s disease

Table 2 shows the baseline comparisons of left and right subcortical structures and the percentage change in volumes between baseline and follow-up, and all *P*-values were uncorrected for the number of subcortical structures (2 × 7). At baseline, the left nucleus accumbens showed significant atrophy in the PD-MCI group compared to PD-NC (*P = *0.05) and at trend level relative to healthy controls (*P = *0.06). The PD-MCI group also had significant atrophy of the left hippocampus relative to PD-NC (*P = *0.03). Over 18 months, the PD-MCI group demonstrated significantly greater atrophy of the left caudate relative to the healthy controls (*P = *0.04). We also found greater atrophy over time in the right hippocampus of the PD-MCI compared to both healthy controls (*P = *0.04) and PD-NC (*P = *0.03). No significant associations were found between subcortical volumes and cognitive tests.

**Table 2:** Comparisons of subcortical volumes at baseline and longitudinal changes over 18 months

Hemisphere	Subcortical segmentations (ml)	Healthy controls		PD-NC		PD-MCI		*P*-value	
		Baseline volume	% change	Baseline volume	% change	Baseline volume	% change	Baseline volume^a^	% change^a^
Left	Thalamus	7.6 ± 0.8	−1.0 ± 4.7	7.9 ± 0.8	−0.2 ± 5.2	7.5 ± 0.8	−0.6 ± 5.1	0.1	0.7
	Caudate	3.4 ± 0.5	1.0 ± 4.9	3.5 ± 0.5	−1.2 ± 4.9	3.6 ± 0.7	−2.5 ± 8.8*	0.7	0.05
	Putamen	4.7 ± 0.6	1.0 ± 7.7	4.8 ± 0.7	−1.2 ± 5.8	4.7 ± 0.7	−2.0 ± 7.1	0.9	0.1
	Pallidum	1.5 ± 0.2	1.3 ± 9.0	1.5 ± 0.2	−0.4 ± 10.3	1.5 ± 0.2	−1.4 ± 8.6	0.7	0.5
	Hippocampus	4.0 ± 0.4	−0.8 ± 5.3	4.2 ± 0.6	−1.5 ± 4.5	3.9 ± 0.4 **	−3.2 ± 6.0	0.04	0.2
	Amygdala	1.4 ± 0.2	−1.8 ± 7.1	1.5 ± 0.2	−0.4 ± 8.1	1.4 ± 0.2	−2.5 ± 6.7	0.8	0.3
	Accumbens	0.4 ± 0.1	−0.9 ± 25.1	0.4 ± 0.1	0.3 ± 23.7	0.37 ± 0.1*	−4.0 ± 22.9	0.04	0.9
Right	Thalamus	6.6 ± 0.7	0.0 ± 4.9	6.9 ± 0.6	−1.4 ± 4.5	6.6 ± 0.8	−1.4 ± 4.0	0.6	0.5
	Caudate	3.1 ± 0.5	−0.4 ± 4.3	3.3 ± 0.4	−2.1 ± 7.3	3.3 ± 0.5	−2.0 ± 6.8	0.4	0.6
	Putamen	4.6 ± 0.6	−0.9 ± 5.3	4.8 ± 0.6	−2.1 ± 7.4	4.6 ± 0.7	−1.4 ± 5.3	0.6	0.7
	Pallidum	1.4 ± 0.1	−2.5 ± 7.0	1.5 ± 0.2	−1.8 ± 8.8	1.4 ± 0.2	−1.3 ± 7.9	0.5	0.8
	Hippocampus	4.1 ± 0.5	−1.7 ± 4.2	4.3 ± 0.5	−1.7 ± 4.7	4.0 ± 0.5	−5.3 ± 8.9***	0.2	0.02
	Amygdala	1.5 ± 0.2	−1.2 ± 11.0	1.6 ± 0.2	−2.2 ± 9.0	1.5 ± 0.2	−7.0 ± 15.4	0.1	0.1
	Accumbens	0.5 ± 0.1	0.3 ± 13.0	0.5 ± 0.1	−1.3 ± 13.2	0.5 ± 0.1	−2.0 ± 13.6	0.1	0.9

Values expressed as mean ± 1 SD.

*PD-MCI < HC, **PD-MCI < PD-NC, *P < *0.05; ***PD-MCI < PD-NC and healthy controls, *P* < 0.05.

^a^ANCOVA = healthy controls, PD-NC, PD-MCI, correcting for age, gender, education, and intracerebral volume.

## Discussion

The key findings in relation to our hypotheses are as follows: (i) at baseline, PD-MCI is characterized by reduced cortical thickness in both frontal and temporo-parietal cortices with atrophy of the nucleus accumbens and the hippocampus; (ii) compared to PD-NC and healthy controls, PD-MCI developed an increased percentage of cortical thinning in frontal and temporo-parietal cortices as well as progressive hippocampal and caudate atrophy over 18 months; (iii) PD-NC cases who converted to PD-MCI over 18 months had temporal cortex thinning at baseline; and (iv) although regional cortical thickness was comparable in the entire PD-NC cohort and healthy controls at baseline, the PD-NC cases subsequently developed significant frontal cortical thinning over 18 months.

Previous cross-sectional imaging studies of cortical thickness in early Parkinson’s disease have yielded inconclusive findings (Zarei *et al.*, 2013; Mak *et al.*, 2014) and earlier longitudinal studies have only assessed global measures such as whole brain atrophy rates (Burton *et al.*, 2005). To date, the only two previous longitudinal studies in cortical thickness have been limited by their relatively small sample size (Hanganu *et al.*, 2014) and heterogeneous Parkinson’s disease cohorts (Ibarretxe-Bilbao *et al.*, 2012). Ibarretxe-Bilbao and colleagues found that non-demented Parkinson’s disease subjects had more progressive cortical thinning than controls with a bilateral fronto-temporal pattern, extending to the parietal cortex (Ibarretxe-Bilbao *et al.*, 2012). Another study reported faster rates of cortical thinning in the frontal and temporal cortices, as well as the insular and supplementary motor areas (Hanganu *et al.*, 2014).

In the present study, we evaluated baseline cortical thickness and subsequently compared the progression of cortical thinning as well as subcortical atrophy over 18 months in a large and well-characterized cohort of non-demented Parkinson’s disease subjects and healthy controls. The present study also extended the literature as our Parkinson’s disease cohort was further classified into PD-MCI and PD-NC based on the formal MDS criteria.

At baseline, PD-NC did not differ significantly from healthy controls in relation to cortical thickness though a subgroup who subsequently converted to PD-MCI showed temporal cortex thinning. Our results are in broad agreement with several previous studies (Dalaker *et al.*, 2010; Ibarretxe-Bilbao *et al.*, 2012; Melzer *et al.*, 2012; Zarei *et al.*, 2013). Other studies have demonstrated reduced cortical thickness in PD-NC compared to healthy controls (Rowe *et al.*, 2010; Pereira *et al.*, 2014). Several reasons may account for these discordant findings in the literature, including heterogeneity in the PD-NC sample, especially in relation to their stage of disease, and variability in the use of different techniques assessing grey matter changes.

Compared to healthy controls, the morphological profile of structural changes was more widespread in PD-MCI subjects with cortical thinning spanning across the frontal, parietal, and occipital cortex as well as the cingulate cortex. This extensive pattern of cortical thinning is in accordance with previous studies (Hanganu *et al.*, 2013; Pereira *et al.*, 2014; Segura *et al.*, 2014). In addition, our results are also consistent with a previous PET study that reported reduced fluorodeoxyglucose (FDG) uptake in the parietal and occipital lobes as well as localized areas of the frontal and temporal lobes in PD-MCI (Garcia-Garcia *et al.*, 2012).

As the implementation of the MDS Task Force diagnostic criteria for PD-MCI in 2012, a limited number of studies have sought to determine its biological validity by determining structural differences in PD-MCI and PD-NC without reaching a consensus: decreased cortical thickness has been reported (Hanganu *et al.*, 2013; Pereira *et al.*, 2014), while others have not observed any difference compared to PD-NC (Mak *et al.*, 2014; Segura *et al.*, 2014). Here, we did not detect any difference in baseline cortical thickness between PD-MCI and PD-NC. Although this could be because early cognitive impairment results from functional deficit before cell loss, it may also be a false negative with PD-NC lying intermediate between healthy controls and PD-MCI.

At the subcortical level, there is accumulating evidence that non-demented Parkinson’s disease is associated with structural changes in the nucleus accumbens (Lee *et al.*, 2014). Another longitudinal study found a significant decrease of grey matter in the nucleus accumbens over time in PD-MCI relative to PD-NC (Hanganu *et al.*, 2014). There is also evidence that atrophy of the nucleus accumbens is predictive of cognitive decline in the elderly (de Jong *et al.*, 2012) and associated with depression (Pizzagalli *et al.*, 2009). In line with our observation of higher depression scores in PD-MCI, previous studies have reported that depression is more common in PD-MCI than PD-NC (Monastero *et al.*, 2013). These findings all suggest that the nucleus accumbens might be a possible subcortical neural substrate for cognitive impairment and neuropsychiatric symptoms in Parkinson’s disease.

To overcome the limitation of past cross-sectional approaches and to clarify previous inconclusive cross-sectional findings of morphological changes in non-demented Parkinson’s disease, the present study specifically investigated regional rates of cortical thinning over 18 months. Despite demonstrating preserved cortical thickness relative to healthy controls at baseline, the PD-NC group subsequently developed significant frontal cortical thinning over the follow-up period. This finding is particularly noteworthy in the absence of cognitive decline and raises two important points: (i) progressive cortical thinning may precede significant cognitive decline; and (ii) compensatory functional mechanisms might be masking or buffering against cognitive decline in our cohort of newly diagnosed PD-NC subjects. Our longitudinal observation is also consistent with previous reports of frontal thinning in PD-NC compared to healthy controls at baseline (Tinaz *et al.*, 2011; Hanganu *et al.*, 2013; Pagonabarraga *et al.*, 2013).

In accordance with our hypothesis, the PD-MCI group showed a greater percentage of cortical thinning in frontal and temporo-parietal areas relative to both PD-NC and healthy controls. These areas are commonly reported to be atrophic in Parkinson’s disease dementia (Burton *et al.*, 2004; Beyer *et al.*, 2007; Hwang *et al.*, 2013). In agreement with our observations of frontal involvement in PD-MCI at baseline and over 18 months, a previous VBM study following a group of PD-MCI subjects over 2 years also found that Parkinson’s disease dementia converters showed increased frontal atrophy compared to non-converters (Lee *et al.*, 2014). Extending beyond the dorsal-lateral prefrontal cortex, the temporo-parietal pattern of cortical thinning demonstrated by the PD-MCI group has also been regarded as a marker of Alzheimer’s disease pathology (Whitwell *et al.*, 2011). As such, considering the transitory stage of PD-MCI within the cognitive spectrum of Parkinson’s disease, it is plausible to suggest that our longitudinal findings are heralding subsequent cognitive deterioration and progression to Parkinson’s disease dementia.

The extended involvement from frontal regions in PD-NC to the temporal regions observed in PD-MCI is also consistent with prevailing theories of neurotransmitter deficits underpinning cognitive dysfunction in Parkinson’s disease. The focal pattern of increased frontal thinning could be associated with concurrent disruption of dopaminergic, serotonergic, cholinergic or noradrenergic frontal-striatal circuits (Hilker *et al.*, 2005; Calabresi *et al.*, 2006; Klein *et al.*, 2010; Politis *et al.*, 2010) as well as cortical deafferentation as a result of white matter change (Rae *et al.*, 2012).

It is worth discussing our longitudinal findings in light of a previous longitudinal cortical thickness study with a similar follow-up duration of 19.9 months (Hanganu *et al.*, 2014). Although our findings are congruent and share the overarching conclusion that PD-MCI is characterized by greater percentage of cortical thinning compared to PD-NC and healthy controls, there were notable differences in both the magnitude and spatial extent of cortical thinning. Firstly, we did not find significant cortical thinning in the occipital regions in PD-MCI, even though reduced cortical thickness of the occipital region was present in PD-MCI at baseline. Secondly, the previous study (Hanganu *et al.*, 2014) did not report any significant difference in frontal regions between groups. Thirdly, fewer peaks in the cortical thickness comparisons survived multiple comparisons. One likely explanation could be the relatively smaller sample size (17 PD-MCI, 15 PD-NC, and 18 healthy controls) that would have limited the statistical power of their cortical thickness analysis to reveal increased thinning in frontal regions in PD-MCI.

Our longitudinal analyses of subcortical structures also revealed significant atrophy of both the caudate nucleus and the hippocampus after 18 months in PD-MCI. Although previous cross-sectional studies have found reduced hippocampal volumes in PD-MCI (Weintraub *et al.*, 2011), our study is the first to report increased progression of hippocampal atrophy in PD-MCI over time. Together, these observations agree with histopathological evidence indicating that the hippocampus is a target for Lewy body inclusions in Parkinson’s disease, with particular vulnerability in the CA2 subfield (Churchyard and Lees, 1997). Interestingly, the caudate nucleus also showed atrophy in the PD-MCI group over 18 months, possibly reflecting dopamine deafferentation and consistent with the role of dopaminergic disruption in cognitive impairment found in Parkinson’s disease. A relationship between caudate dopamine transporter (DAT) binding and cognition has been reported (Polito *et al.*, 2012) and longitudinal studies have demonstrated that reduced baseline caudate DAT can predict subsequent cognitive decline (Ravina *et al.*, 2012). The caudate nucleus, receiving dopaminergic inputs from the substantia nigra, is interconnected with the dorsolateral prefrontal cortex (Leh *et al.*, 2007). Thus, through these neuronal loops, dopaminergic deficits could indirectly impair frontal functions. Considered together, both our longitudinal subcortical findings in PD-MCI confer support to a previous cross-sectional finding that impaired cognition is related to caudate dopaminergic hypofunction as well as hippocampal atrophy (Jokinen *et al.*, 2009).

In summary, our joint investigation of baseline and longitudinal MRI measures in a well-delineated Parkinson’s disease cohort allows us to integrate the findings into a temporal and severity-gradient framework: at the time of Parkinson’s disease diagnosis, frontal-striatal deficits are already prominent in PD-NC. Subsequently, the deterioration of cognitive function to PD-MCI is associated with an extension from frontal regions to a wider involvement of posterior temporal cortices, most probably as a result of its multifactorial nature in conjunction with generalized disruptions of other neurotransmitter systems. In addition, we propose that the focal thinning in frontal regions causes a progressive degeneration of the reciprocal cortico-cortical connections between the temporal and frontal regions (Simons and Spiers, 2003). This notion is supported by our finding of additional temporal cortical thinning in PD-MCI. Furthermore, these patterns of cortical thinning, particularly the differential involvement of posterior cortical regions in PD-MCI, are also consistent with the neuropsychological evolution demonstrated in a different incident cohort of patients with Parkinson’s disease by the CamPaIGN study (Williams-Gray *et al.*, 2009). Our study also provides structural imaging support for the dual-syndrome hypothesis, which posits that the frontostriatal deficits relate to dopaminergic deficits and that the development of dementia is associated with a more widespread and posterior pattern of cortical changes (Kehagia *et al.*, 2012). Finally, we noted an asymmetric pattern of cortical thinning in the left hemisphere of the PD-NC relative to healthy controls over 18 months. This finding suggests that cortical changes could start initially in one hemisphere and subsequently extend to the other as cognitive capacity deteriorates, as observed by our bilateral involvement in PD-MCI.

The main strengths of this prospective cohort study are the longitudinal design of an incident cohort, which enables the investigation of progressive structural changes in Parkinson’s disease, a topical area of research where current knowledge is limited. Our sample size was larger than in most previous series and all subjects undertook comprehensive neuropsychological evaluation. In addition, our findings were robust after correction for multiple comparisons and accounting for effects of age, which is necessary given its intimate association with cortical thinning (Salat *et al.*, 2004) but often overlooked in previous studies.

Several limitations should also be recognized. As with all ante-mortem studies, we did not have neuropathological confirmation of Parkinson’s disease. To further protect against potential misdiagnosis, subjects were reassessed after 18 months, revealing no change in diagnoses. It should also be noted that subjects were assessed while taking their medication, which could influence cognition and cortical thickness. The Parkinson’s disease subjects showed a counter-intuitive improvement in MoCA at follow-up. This might reflect dopaminergic modulation of executive function, which appears to be highly involved in some of the subtests of the MoCA. Indeed, there have been previous studies showing that executive function may be improved by levodopa (Mattay *et al.*, 2002). However, assessing patients during their ON state is in keeping with current clinical practice where many patients are treated with antiparkinsonian medication following diagnosis if symptom severity dictates the need to do so. Furthermore, this will equate to a best ON-state during longitudinal assessment. Lastly, unlike previous studies, we have accounted for variance due to levodopa equivalent daily dose in our imaging analyses.

Although the neuropsychological battery in the present study has been previously described in detail (Yarnall *et al.*, 2014), our assessment of visuospatial function was limited as we only had one representative test (i.e. pentagon copying item of the MMSE). However, in recent years, the pentagon copying item of the MMSE has received increasing recognition in its predictive value of dementia in Parkinson’s disease from several population-based longitudinal studies, suggesting that dementia is heralded by posterior-based cognitive deficits (Williams-Gray *et al.*, 2007, 2009). A recent study has also identified imaging correlates of the pentagon copying test in a cohort of Parkinson’s disease subjects in bilateral posterior regions, such as the temporo-parietal cortices (Garcia-Diaz *et al.*, 2014). The inclusion of an additional test of visuospatial ability would be beneficial in future studies.

Finally, the dichotomization of non-demented Parkinson’s disease subjects into PD-MCI and PD-NC is not without potential limitations. A community-based cohort of 159 newly diagnosed patients with Parkinson’s disease (CamPaIGN study) revealed deficits in frontostriatal-based tasks (12%), temporal lobe-based tasks (8%), and global cognition (15%) (Foltynie *et al.*, 2004). Given the near ubiquitous nature and heterogeneity of cognitive deficits in Parkinson’s disease, the relative importance of various cognitive profiles in the development of Parkinson’s disease dementia remains a topic of continuing debate. Although executive deficits and attention have been implicated in the development of Parkinson’s disease dementia (Janvin *et al.*, 2005; Pedersen *et al.*, 2013), a 3.5-year follow-up of the CamPaiGN cohort further clarified the evolution of cognitive deficits in Parkinson’s disease by showing that cognitive deficits with a posterior cortical basis (i.e. semantic fluency and visuospatial ability) are most associated with progressive global decline (Williams-Gray *et al.*, 2007). However, the use of the MDS criteria in the present literature precludes a finer delineation of PD-MCI into two subgroups: one that is frontal-striatal based and another that is posterior cortical based with greater risks of dementia in Parkinson’s disease.

In conclusion, our combined baseline and follow-up analyses revealed that PD-MCI is associated with widespread reductions of cortical thickness at baseline accompanied by a more severe progression of cortical thinning. In particular, the frontal and temporal cortical thinning in PD-MCI may be predictive of progression to Parkinson’s disease dementia. This study provides strong evidence that structural MRI can aid early detection of cortical involvement in Parkinson’s disease and target appropriate intervention.

## Supplementary Material

Supplementary Table 1

## Abbreviations

MDS: Movement Disorders Society
MMSE: Mini-Mental State Examination
MoCA: Montreal Cognitive Assessment
PD-MCI: Parkinson’s disease with mild cognitive impairment
PD-NC: Parkinson’s disease with no cognitive impairment
UPDRS: Unified Parkinson’s Disease Rating Scale
