# Supplementary material for: Baseline and longitudinal grey matter changes in newly diagnosed Parkinson’s disease: ICICLE-PD study
Source: Brain. 2015 Jul 14;138(10):2974–86. doi: 10.1093/brain/awv211 (PMC4671477; doi:10.1093/brain/awv211)
Supplement: Supplementary Table 1 [file brain_awv211_index.html]

Supplementary Data | Brain

## Supplementary Data

files

- Supplementary Data - pdf file
- Supplementary Data - png file
