# Supplementary material for: Baseline and longitudinal grey matter changes in newly diagnosed Parkinson’s disease: ICICLE-PD study
Source: Brain. 2015 Jul 14;138(10):2974–86. doi: 10.1093/brain/awv211 (PMC4671477; doi:10.1093/brain/awv211)
Supplement: Supplementary Table 1 [file f822f9bdb421e3133935d150b75ea541_brain-2015-00555-File002.pdf]

SUPPLEMENTARY MATERIAL

Cognitive domains and sub-tests	Healthy controls	PD-NC	PD-MCI
**Attention**			
PoA	1247.6 ± 123.3	1285.9 ± 114.8	1442.0 ± 250.4
Digit vigilance accuracy (%)	96.5 ± 6.7	97.2 ± 4.2	85.6 ± 15.5
**Memory**			
PRM	21.3 ± 1.9	21.3 ± 2.0	17.7 ± 3.1
SRM	16.8 ± 1.6	16.2 ± 1.7	13.8 ± 2.6
PAL	1.6 ± 0.5	1.7 ± 0.4	2.5 ± 1.0
**Executive function**			
One touch stockings	16.4 ± 2.3	16.0 ± 2.2	12.2 ± 4.8
Phonemic fluency	12.7 ± 4.8	12.4 ± 4.4	9.1 ± 3.9
Semantic fluency	24.5 ± 6.6	23.3 ± 5.5	17.8 ± 6.4
**Visuospatial**			
Pentagon copying	1.9 ± 0.3	2.0 ± 0.1	1.7 ± 0.5
**Language**			
Naming	2.9 ± 0.3	2.9 ± 0.3	2.9 ± 0.4
Sentence	1.8 ± 0.5	1.8 ± 0.5	1.5 ± 0.7

Supplementary Table 1. Cognitive profiles of subjects with PD according to cognitive domains in the MDS criteria. Abbreviations: PD-MCI = Parkinson’s disease with mild cognitive impairment; PoA = Power of Atttention; PRM = Pattern Recognition Memory; SRM = Spatial Recognition Memory; PAL = Paired Associates Learning.

Anatomical region	Talairach coordinates X	Y	Z	No. of vertices	Cluster size (mm^2)	$-log_{10}P$
**(i) PD-MCI < Healthy controls**						
L supramarginal	-49.6	-48.0	44.6	13586	7400.02	-4.00
L rostral middle frontal	-38.1	50.0	-3.4	6098	3299.31	-4.00
L isthmus cingulate	-4.1	-33.4	30.5	4552	2245.71	-2.49
R rostral middle frontal	21.1	60.3	6.1	6246	3893.08	-4.00
R lateral occipital	21.3	-98.7	5.3	5724	3745.91	-4.00
R posterior cingulate	14.0	-18.1	38.4	5680	2359.30	-2.41

Supplementary Table 2. Regions showing significantly reduced cortical thickness in PD-MCI compared to healthy controls at baseline. Age, sex, and education were included as covariates. The anatomical region, ($-log_{10}$) p value, and Talairach coordinates correspond to the most significant vertex within each cluster. Abbreviations: PD-MCI = Parkinson's disease with mild cognitive impairment.

Anatomical region	Talairach coordinates X	Y	Z	No. of vertices	Cluster size (mm^2)	$-log_{10}P$
**(i) PD < Healthy controls**						
L caudal middle frontal	-41.4	3.5	46.8	3076	1534.48	-2.64
**(ii) PD-MCI < Healthy controls**						
L superior frontal	-11.5	-7.7	47.3	21345	11029.66	-4.00
L supramarginal	-49.6	-48.0	44.6	14557	6654.22	-4.00
R precuneus	9.4	-50.8	47.8	13433	6245.25	-4.00
**(iii) PD-MCI < PD**						
L caudal middle frontal	-30.8	9.3	53.6	4771	2407.01	-4.00
L superior temporal	-61.4	-48.1	15.5	3208	1407.12	-2.10
R superior frontal	11.0	14.6	62.2	4863	2102.10	-3.52

Supplementary Table 3. Regions showing significant percentage change of cortical thickness over 18 months (i) PD vs Healthy controls, (ii) PD-MCI vs Healthy controls, (iii) PD-MCI vs PD. Age, sex, and education were included as nuisance covariates while LEDD was an additional covariate in the comparison between PD-MCI and PD-NC. The anatomical region, ($-log_{10}$) p value, and Talairach coordinates correspond to the most significant vertex within each cluster. Abbreviations: PD = Parkinson’s disease; PD-MCI = Parkinson’s disease with mild cognitive impairment; LEDD = Levodopa Equivalent Daily Dosage.

**Group – Cognitive test**	**Anatomical region**	**Talairach coordinates**			**No. of vertices**	**Cluster size (mm^2)**	**$-log_{10}P$**
		**X**	**Y**	**Z**			
All PD – MoCA	L fusiform	-42.5	-64.6	-18.8	5711	3682.67	4.0000
	L superior frontal	-11.5	-7.7	47.3	6544	3217.14	3.3979
	L inferior parietal	-41.3	-67.3	27.3	5571	2643.99	2.6383
	L lateral orbitofrontal	-26.3	23.8	-6.0	5634	2247.28	2.0862
	R parahippocampal	31.0	-41.1	-9.0	4068	2352.95	2.2147

Supplementary Table 4. Correlations between regional cortical thickness and global cognition at baseline. Age, sex, and education were included as covariates. The anatomical region, ($-log_{10}$) p value, and Talairach coordinates correspond to the most significant vertex within each cluster. Abbreviations: PD = Parkinson's disease; PD-MCI = Parkinson's disease with mild cognitive impairment.
