# Supplementary figures and images for: Baseline and longitudinal grey matter changes in newly diagnosed Parkinson’s disease: ICICLE-PD study
Source: Brain. 2015 Jul 14;138(10):2974–86. doi: 10.1093/brain/awv211 (PMC4671477; doi:10.1093/brain/awv211)

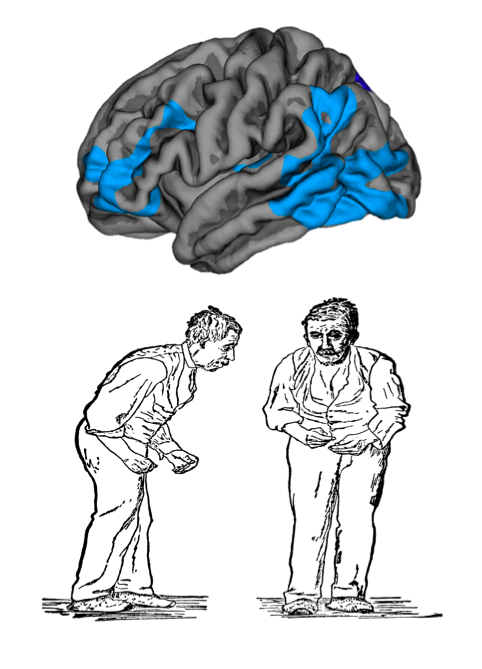

Supplement: Supplementary Table 1 [file 2c1f7d11345b5cb15bdaea86b741889f_brain-2015-00555-File003.png]
